# Formulation and Characterization of Oxiconazole-Loaded Novasomes to Enhance the Treatment of Fungus Infections: Experimentally Induced Candidiasis in Rat

**DOI:** 10.3390/ph18121803

**Published:** 2025-11-26

**Authors:** Ibrahim A. Mousa, Abdelghafar M. Abu-Elsaoud, Shereen A. Sabry, Mahmoud Abd Elghany, Dina Khodeer, Fathy E. Abdelgawad, Ali M. Nasr

**Affiliations:** 1 General Authority of Health Care, Ismailia Governorate, Ismailia 11517, Egypt; 2 Biology Department, College of Science, Imam Mohammad Ibn Saud Islamic University (IMSIU), P.O. Box 1690950, Riyadh 11623, Saudi Arabia; 3 Department of Pharmaceutics, Faculty of Pharmacy, Zagazig University, Zagazig 44519, Egypt; 4 Department of Pharmacology, College of Medicine, Imam Mohammad Ibn Saud Islamic University (IMSIU), Riyadh 13317, Saudi Arabia; 5 Department of Chemistry, Faculty of Science, Islamic University of Madinah, Madinah 42351, Saudi Arabia; 6 Department of Pharmaceutics and Industrial Pharmacy, Faculty of Pharmacy, Galala University, Suez 43713, Egypt

**Keywords:** oxiconazole, novasomes, topical delivery, ethanol injection technique, Box–Behnken design, antifungal efficacy

## Abstract

**Background/Objectives**: An observed increase in fungal infection incidence over the past two decades underscores the limitations of conventional topical treatments for deep infections, primarily due to the skin’s stratum corneum barrier. This has driven the development of advanced topical preparations. This study evaluated the encapsulation of oxiconazole utilizing novasomes to enhance its topical delivery. **Methods**: Oxiconazole-loaded novasomes were synthesized by the ethanol injection technique and subsequently characterized using key physicochemical parameters, including encapsulation efficiency (EE%), vesicle size (VS), zeta potential (ZP), polydispersity index (PDI), and percentage drug release (DR%). The optimized formulation underwent comprehensive evaluation employing Fourier transform infrared spectroscopy (FTIR), differential scanning calorimetry (DSC), and transmission electron microscopy (TEM). Moreover, its activity was evaluated through in vitro penetration studies and in vivo assessments. **Results**: R9 was identified as the optimal candidate, demonstrating an encapsulation efficiency of 94.63 ± 1.60%, a vesicle size of 174 ± 1.15 nm, a zeta potential of −46.5 ± 1.61 mV, a polydispersity index of 0.184 ± 0.01, and a drug release rate of 51 ± 0.50% within 8 h. This optimal formula achieved 94 ± 1.75% permeation of oxiconazole within 24 h. FTIR examination affirmed the interaction of oxiconazole and the excipients, while DSC analysis verified the thermal durability of oxiconazole. In vivo histopathological examination demonstrated the superior efficacy of the optimal formula in treating *Candida albicans* infection. **Conclusions**: Novasomes emerge as a promising and efficacious system for oxiconazole encapsulation, holding significant potential for the effective and prolonged management of topical fungal infections.

## 1. Introduction

Fungal diseases classified as superficial mycoses primarily affect the skin, nails, and mucous membranes, and they represent some of the most frequently encountered pathogens causing cutaneous damage. The global incidence of these infections is reportedly on the rise, with recent estimates indicating approximately 6.5 million new cases each year and 3.8 million related deaths across the globe [1]. *Candida albicans* constitutes the most frequently encountered fungal species and is recognized as a significant etiological factor in the development of superficial and systemic fungal infections [2].

Oxiconazole nitrate is an azole-class antifungal agent utilized in the management of various pathogenic fungus diseases, including those attributed to *Candida albicans* and *Tinea* species. It is characterized by an intermediate molecular weight, low potential for skin irritation, and favorable skin permeability, making it suitable for topical therapeutic applications [3]. Its antifungal efficacy arises from the inhibition of ergosterol biosynthesis, a process critical for preserving the structural solidity and functionality of the fungal cell membrane [4]. However, currently available oxiconazole formulations have been linked to local adverse reactions—such as erythema, irritation, and allergic contact dermatitis [3].

Treatment approaches for fungal infections involve topical and systemic antifungal therapy. Topical formulations are often favored because of the potential drawbacks associated with systemic therapy, such as organ-specific toxicity, significant drug–drug interactions, and increased healthcare costs [5].

Nevertheless, the stratum corneum, the skin’s outermost layer, serves as the primary barrier to drug permeation. Consequently, to achieve adequate absorption and therapeutic efficacy for antifungal agents, it is crucial to produce an advanced drug delivery system in order to effectively bypass or overcome this barrier [6,7]

Topical and transdermal routes are often favored over systemic administration due to their ease of use and enhanced patient acceptance. Nevertheless, traditional topical formulations like creams and gels are generally inadequate for effectively treating skin fungal infections, especially those that are deep-seated. This is primarily because they lack the necessary capacity to penetrate the skin barrier [8].

Moreover, conventional formulations are typically prepared for rapid drug release and may provoke immune responses, potentially leading to severe allergic reactions. Consequently, there is a critical demand to formulate drug delivery systems that incorporate drugs in nanoparticulate form while also enabling sustained release to improve the effectiveness of therapy and mitigate undesirable actions [9].

Several advanced vesicular systems, including transfersomes and ethosomes, have been developed to overcome the restrictions of traditional vesicles, like liposomes and niosomes, which often exhibit inadequate skin penetration and suboptimal drug distribution. In addition to formulating more effective delivery vehicles for highly potent antifungal agents, the increasing and often indiscriminate use of antimycotic drugs has resulted in the occurrence of resistant fungal strains. This growing resistance underscores the urgent therapeutic need to formulate antifungal agents with innovative delivery systems to enhance treatment outcomes [10].

Novasomes, an advanced encapsulation technology developed by Novavax and IGI Laboratories, were designed to address several limitations associated with conventional drug delivery systems. Defined as modified liposomes or specialized niosomes, novasomes are composed of a monoester of polyoxyethylene fatty acids, cholesterol, and free fatty acids. This unique composition imparts enhanced stability, drug loading capacity, and delivery efficiency, positioning novasomes as a promising platform for improved therapeutic outcomes [10]. Novasomes possess several key characteristics, including a multi-bilayer vesicular structure with a substantial inner core capacity within a defined size range. With an improved encapsulation process and higher entrapment efficiency, novasomes facilitate controlled drug release and reduce dosage frequency [11]. This design enables them to deliver a substantial quantity of active ingredients. Novasome nano-vesicles exhibit inherent stability and are designed to withstand a wide range of temperatures [12]. As vesicles loaded with fatty acids, novasomes are believed to be highly effective at permeating both the nasal membrane and the skin, which enhances the transdermal delivery of encapsulated medications [13].

The primary objective of the current research was to formulate and characterize oxiconazole-loaded novasomal formulations using a Box–Behnken optimization approach and to identify the most effective formulation based on critical quality attributes. Moreover, this study primarily seeks to improve the drug’s ability to permeate the skin, extend its release, enhance its therapeutic outcomes, and support patient compliance by integrating it into a biocompatible and stable transdermal delivery system. The study also aimed to identify the most effective formulation for evaluating its topical antifungal efficacy against an experimentally induced *Candida albicans* infection.

## 2. Results

### 2.1. Preparation of Novasomes and Experimental Design

**Figure 1:**
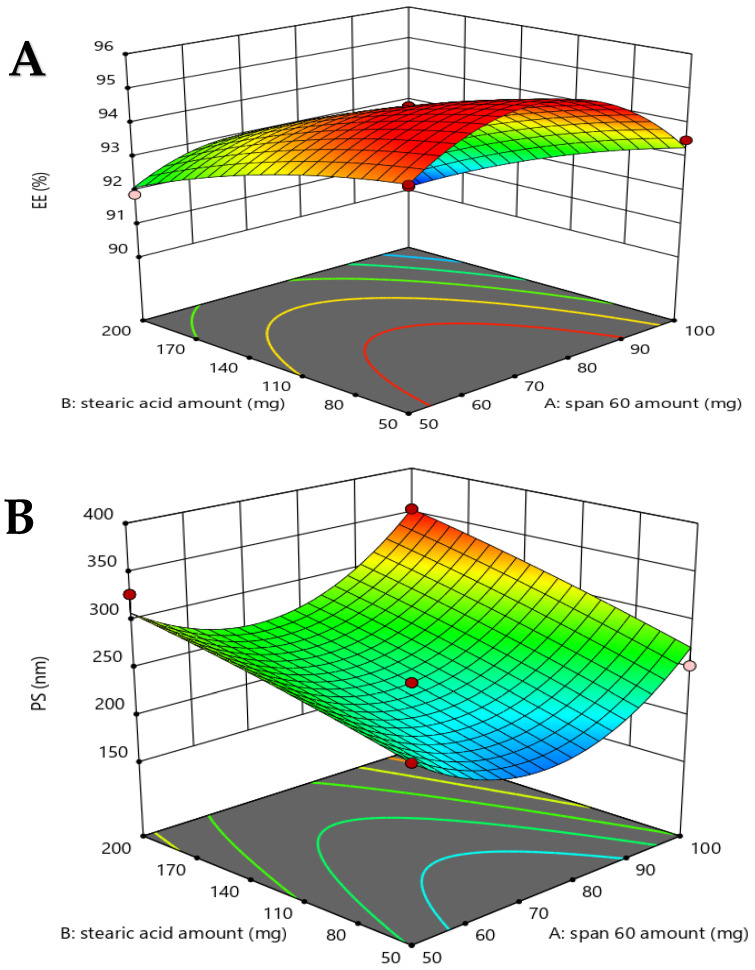

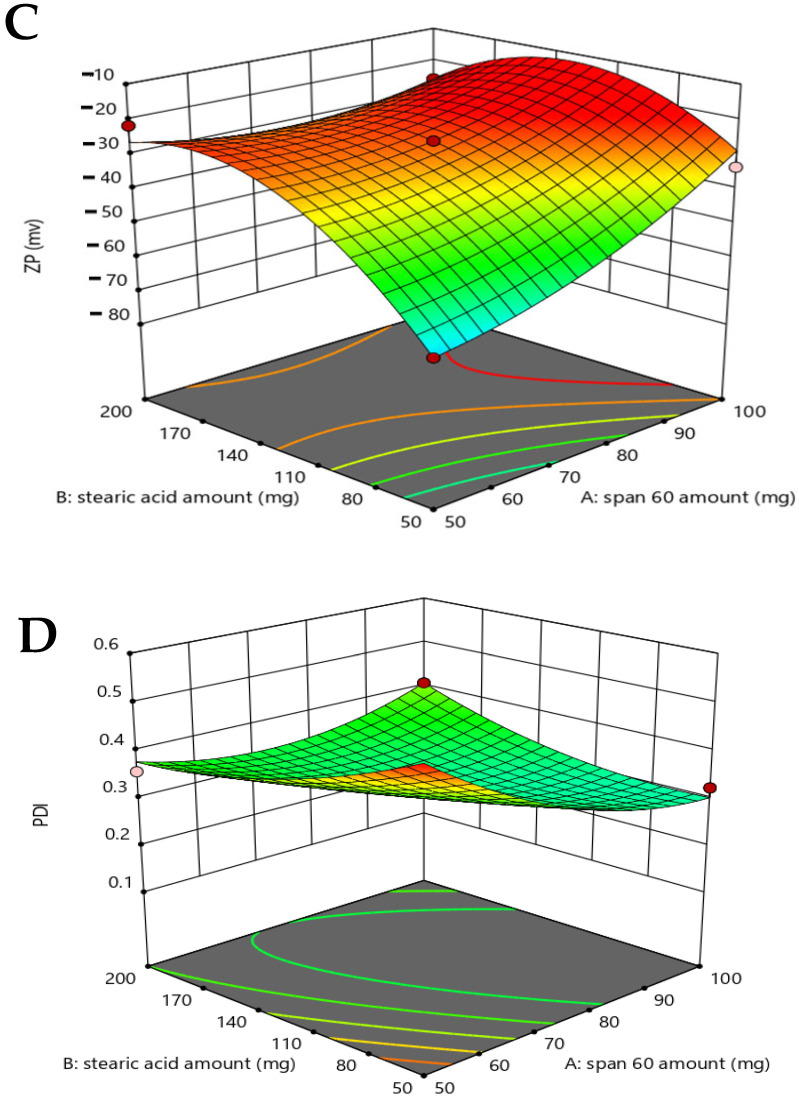

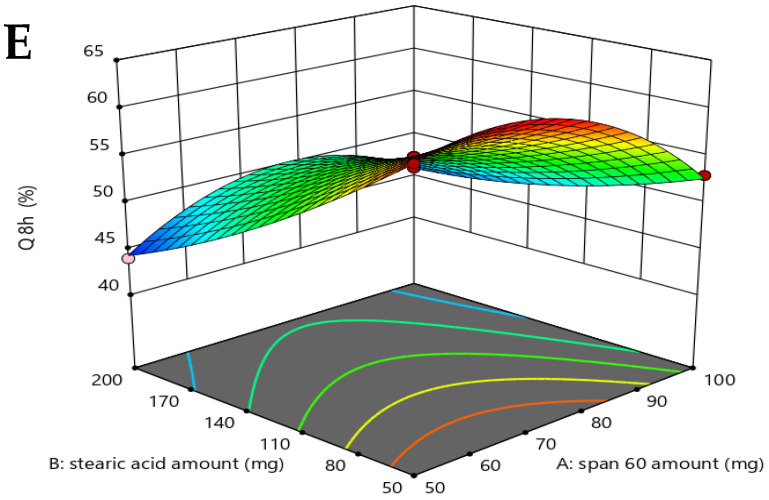
Graphs of three-dimensional response obtaining the effect of the independent parameters on (**A**) EE%, (**B**) VS, (**C**) ZP, (**D**) PDI, and (**E**) DR % 8 h.

The effect of several formulation parameters, specifically the concentrations of Span 60, stearic acid, and cholesterol, was systematically assessed. This evaluation was performed by examining their effects on the encapsulation efficiency percentage (EE%), vesicle size (VS), zeta potential (ZP), polydispersity index (PDI), and percentage of drug release (DR%). The results corresponding to the 17 experimental runs are presented in Table 1 and Figure 1.

### 2.2. Characterization of the Oxiconazole-Loaded Novasomes

#### 2.2.1. Evaluation of Encapsulation Efficacy (EE%)

In the current investigation, the encapsulation efficiency (EE%) of oxiconazole within the novasomal formulations ranged from 86.4 ± 4.8% to 94.63 ± 1.6% (Table 1).

(Figure 1A) visually depicts the response surface graph, illustrating the cumulative effect of two independent parameters on the encapsulation efficacy percentage (%) of oxiconazole-loaded novasomes, with the third parameter held at its middle level. The EE% data demonstrated an excellent fit to a statistically significant quadratic model (*p* < 0.0001) (Table 2). The relationship between the three independent factors and encapsulation efficiency (EE%, Y_1_) was described by the regression equation generated in coded values as in Equation (1).

EE % (Y_1_) = + 94.51 + 0.74 A + 1.33 B + 1.95 C + 0.27 AB + 1.52 AC + 0.0667 BC − 1.76 A^2^ − 0.312 B^2^ − 0.4792 C^2^ (1)

A positive coefficient preceding a factor in the regression equation indicates a direct relationship, meaning that a rise in that parameter’s value leads to a rise in the response, and conversely, a negative coefficient signifies an inverse relationship, where a rise in the parameter’s value results in a reduction in the response [14].

An elevated ratio or concentration of Span 60 (X_1_, Equation (1)A) showed a statistically significant increase (*p* < 0.001) in EE%. Stearic acid (X_2_, Equation (1)B) has a significant positive effect (*p* < 0.001) on encapsulation efficiency (EE%). On the other hand, an increase in the concentration of cholesterol (X_3_, Equation (1)C) results in a statistically significant increase (*p* < 0.001) in EE%.

#### 2.2.2. Assessment of Formulation Parameters on Vesicle Size

Vesicle size (VS) is a crucial indicator of drug permeation through the skin, with nano-sized delivery systems generally facilitating enhanced penetration into deeper skin layers [15]. The VS of the oxiconazole-loaded novasomal formulations varied from 174 ± 1.15 nm to 387.3 ± 49.02 nm, as detailed in Table 1 and visually represented in Figure 1B. The PS data demonstrated statistical significance (*p* < 0.0178) (Table 2). The relationship between the three independent factors and vesicle size (VS: Y_2_) is described by the regression equation generated in coded values, as shown in Equation (2).

PS (Y_2_) = + 235 + 17.97 A + 35.93 B − 72.85 C + 4.80 AB + 52.38 AC + 0.617 BC + 63.70 A^2^ − 3.70 B^2^ − 1.97 C^2^ (2)

The outcomes indicated a non-significant elevation (*p* < 0.12) in the vesicle size (VS) of the fabricated vesicles with rising Span 60 (X_1_, Equation (2)A) concentration, while stearic acid (X_2_, Equation (2)B) showed a significant elevation in vesicle size (*p* < 0.001). On the other hand, cholesterol (X_3_, Equation (2)C) was hypothesized to significantly reduce (*p* < 0.002) vesicle size.

#### 2.2.3. Assessment of Formulation Parameters on Zeta Potential and PDI Analysis

Zeta potential (ZP) is a crucial parameter of the colloidal durability of a nano-system, reflecting the net electrical charge of the dispersed vesicles. Generally, a system exhibiting a ZP value approximately ± 30 mV is considered stable because of sufficient electrostatic repulsion preventing vesicle aggregation [16]. In the current study, the zeta potential values varied from −21.7 ± 0.2 mV to −73.8 ± 0.87 mV, as revealed in Table 1 and Figure 1C.

The ZP outcomes were optimally suitable for a quadratic model, which demonstrated statistical significance (p<0.0036) (Table 2). The specific regression equation for ZP (Y_3_) is provided in Equation (3).

ZP (Y3) = − 26 + 8.38 A + 9.52 B + 9.80 C − 8.05 AB + 4.88 AC + 25.18 BC − 5.27 A^2^ − 14.93 B^2^ − 25.71 C^2^ (3)

It was noted that the zeta potential (ZP) value significantly increased (*p* < 0.012) with increasing amounts of stearic acid (X_2_, Equation (3)B) (*p* < 0.017), Span 60 (X_1_, Equation (3)A) (*p* < 0.01), and cholesterol (X_3_, Equation (3)C) (*p* < 0.028).

The polydispersity index (PDI) results obtained varied from 0.184 ± 0.014 to 0.49 ± 0.14, as shown in Table 1 and Figure 1D.

The PDI data exhibited an optimal fit to a quadratic model, which revealed statistical significance (*p* < 0.0016) (Table 2). The specific regression equation for PDI (Y_4_) is provided in Equation (4).

PDI (Y4) = + 0.32 − 0.049 A − 0.012 B − 0.015 C + 0.064 AB − 0.0038 AC − 0.091 BC + 0.0527 A^2^ + 0.033 B^2^ − 0.071 C^2^ (4)

#### 2.2.4. Release of Oxiconazole from Novasomes

**Figure 2:**
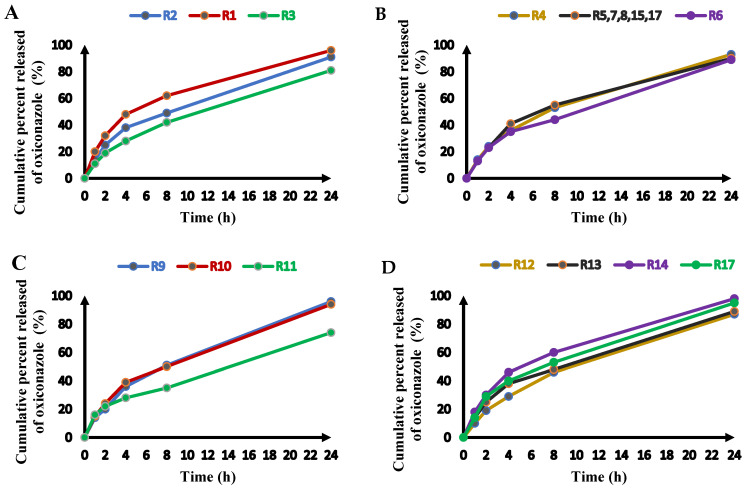
Oxiconazole release from novasomal formulations (Runs) over 24 h divided into (**A**) R1, R2, and R3 (**B**) R4, R5,7,8,15,17 (center points) and R6 (**C**) R9, R10, and R11 (**D**) R12, R13, R14 and R17.

The drug release percentage of oxiconazole from the loaded novasomal preparations ranged from 35 ± 3.20% to 62 ± 1.90% after 8 h (Table 1, Figure 1E) and from 74 ± 2.5% to 98 ± 0.5% after 24 h (Figure 2). The drug release results were best-matched to a quadratic model, which demonstrated high statistical significance (*p* < 0.0001), as shown in Table 2. The influence of the three independent parameters on the drug release percentage (Y_5_), expressed in terms of coded results, is revealed by Equation (5).

DR% 8 h (Y5) = + 55 − 1.92 A − 6.08 B − 1.25 C + 2.75 AB − 0.41 AC − 0.41 BC − 5.13 A^2^ − 1.38 B^2^ − 0.458 C^2^ (5)

The results demonstrated an unfavorable, yet statistically significant, negative influence of Span 60 concentration (X_1_, Equation (5)A) on the 8 h drug release percentage (*p* < 0.001). It was evident that the stearic acid (X_2_, Equation (5)B) content had significant antagonistic consequences on the 8 h drug release percentage (DR% 8 h) of oxiconazole-loaded novasomes (*p* < 0.001). The cholesterol (X_3_, Equation (5)C) content significantly and negatively impacted the 8 h drug release percentage (DR% 8 h) from the novasomal vesicles (*p* < 0.002).

The AB (span 60 and stearic acid) interaction exhibited a positive synergistic effect on encapsulation efficiency (EE %) and vesicle size (VS). The BC (stearic acid and cholesterol) interaction demonstrated a significant effect on EE % and drug release (DR %) [12]. The A^2^ (span 60), B^2^ (stearic acid), and C^2^ (cholesterol) terms indicated curvature in the response surfaces, confirming a non-linear relationship and justifying the quadratic model selection [17].

### 2.3. Selection of the Optimized Formula

The oxiconazole novasomal systems were created, analyzed, and optimized by utilizing the Box–Behnken design. This particular design was chosen because it requires a restricted number of trials to achieve optimization, making it an efficient approach [18]. The statistical analysis performed by the Box–Behnken design demonstrated the mathematical model’s capability in accurately assessing the significant effects of the independent parameters on the measured responses. Furthermore, the adequate precision value, which represents the signal-to-noise ratio, exceeded the threshold value of 4, indicating a strong model signal relative to background noise. This result verifies the model’s reliability and its suitability for effectively navigating and interpreting the experimental design space [19].

**Figure 3:**
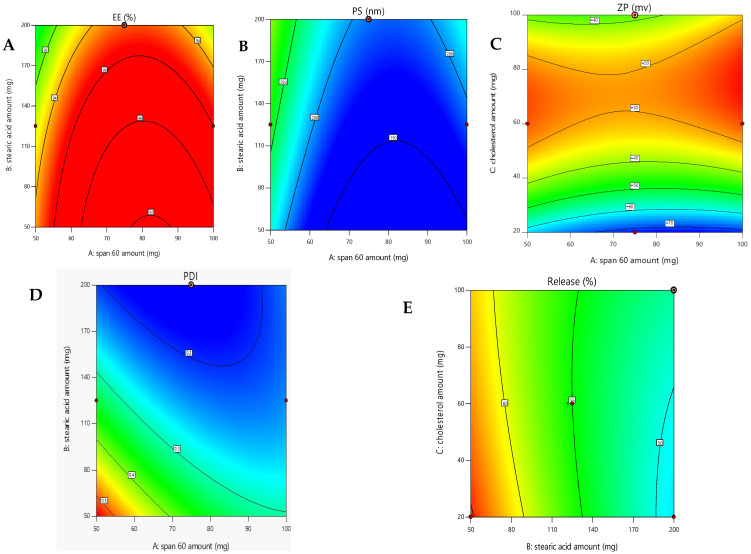
Contour plots of optimized formula (**A**) Encapsulation Efficiency %, (**B**) Vesicle Size, (**C**) Zeta Potential, (**D**) Polydispersity Index, and (**E**) Drug Release %.

The optimized formulation (R9) for the oxiconazole novasomal system was selected using the numerical point anticipating optimized strategy within Design-Expert^®^ model. This selection was based on specific criteria: achieving maximum encapsulation efficiency (EE%) (Figure 3A), minimum vesicle size (VS) (Figure 3B), appropriate zeta potential (ZP) (Figure 3C), narrow polydispersity index (PDI) (Figure 3D), and prolonged drug release (DR%) (Figure 3E), while simultaneously ensuring minimum vesicle size (VS).

Based on the numerical optimization generated using Design-Expert^®^ software (Version 11; Stat-Ease Inc., Minneapolis, MN, USA), an optimal oxiconazole novasomal preparation was recommended with an overall desirability of 0.89. The optimal composition was determined to be 93.53 mg of cholesterol, 79.2 mg of Span 60, and 200 mg of stearic acid.

To validate the model’s predictions, this optimized preparation was made three times, and its dependent results were experimentally evaluated. The optimized formulation (R9) revealed an encapsulation efficiency (EE%) of 94.63 ± 1.6%, a vesicle size (VS) of 174 ± 1.15 nm, a zeta potential (ZP) of −46.5 ± 1.61 mV, a polydispersity index (PDI) of 0.184 ± 0.014, and an 8 h drug release percent (DR%) of 51 ± 0.5%. These characteristics collectively indicate good stability for the formulation.

### 2.4. Thermal Analysis Optimized Oxiconazole Formula

**Figure 4:**
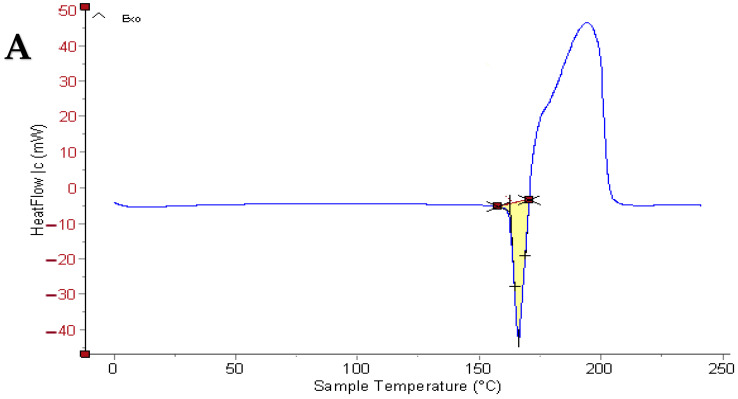

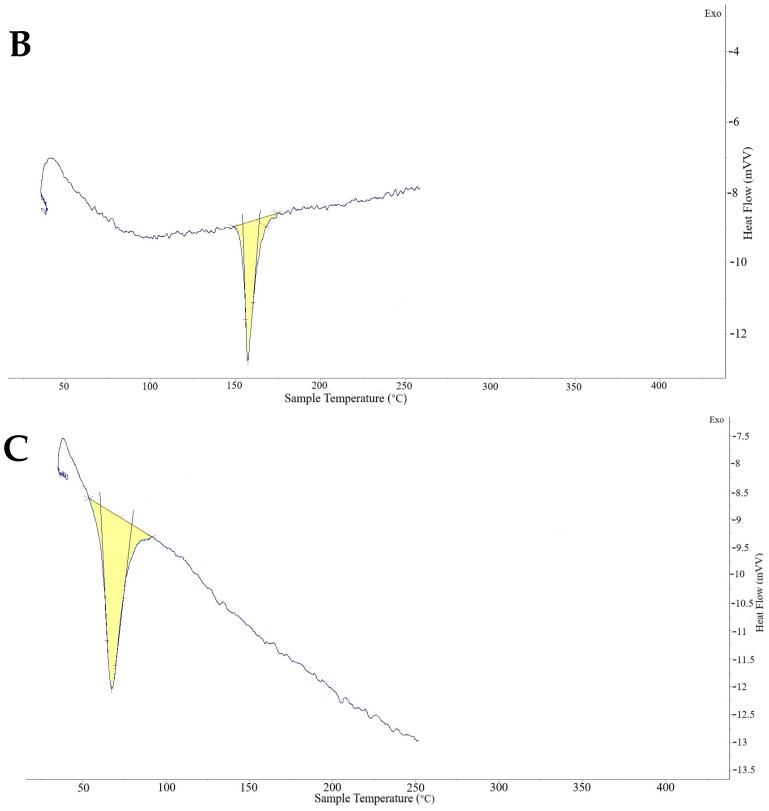

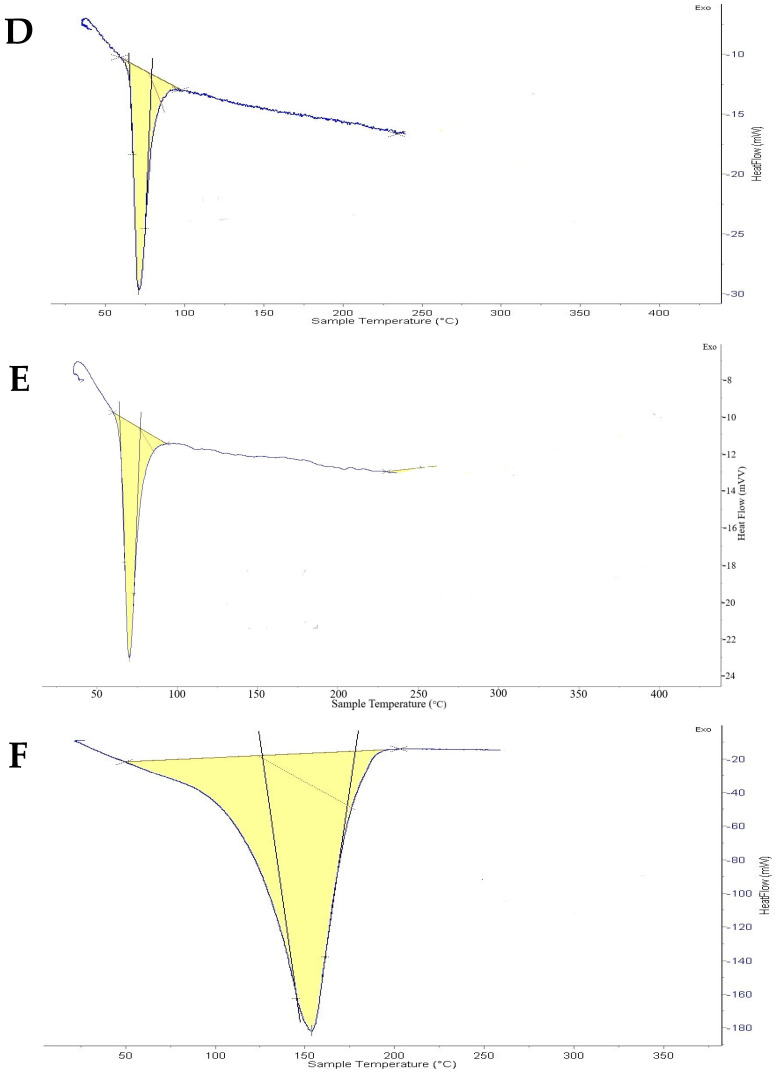
Differential scanning calorimetry observations of (**A**) pure oxiconazole, (**B**) cholesterol, (**C**) span 60, (**D**) stearic acid, (**E**) physical mixture, and (**F**) optimal oxiconazole formula.

Differential Scanning Calorimetry (DSC) is employed to investigate potential physical and chemical interactions between excipients and active pharmaceutical ingredients. Figure 4 illustrates the characteristic endothermic peaks observed for all individual components of the novasomal formulation, physical mixture, and the optimal preparation.

Specifically, oxiconazole revealed a distinct endothermic peak at 166.1 °C (Figure 4A). Cholesterol exhibited an endothermic peak at 157.3 °C (Figure 4B), while Span 60 (Figure 4C) and stearic acid (Figure 4D) presented endothermic peaks at 67.27 °C and 71.18 °C, respectively. The physical mixture of these components displayed a single endothermic peak at 71.1 °C (Figure 4E). Notably, the optimized oxiconazole novasomal formulation (Figure 4F) revealed an endothermic peak at 153.71 °C.

The observed absence of the melting endotherm corresponding to oxiconazole suggests that the drug is present in a more soluble, amorphous form within the novasomal formulation. This alteration in the melting behavior is likely because of the suppression of oxiconazole crystallization and its efficient solubilization within the novasomes. These findings collectively indicate that oxiconazole is homogeneously distributed within novasomes in an amorphous form, which may enhance its dissolution rate and bioavailability [13].

### 2.5. FTIR Analysis

**Figure 5:**
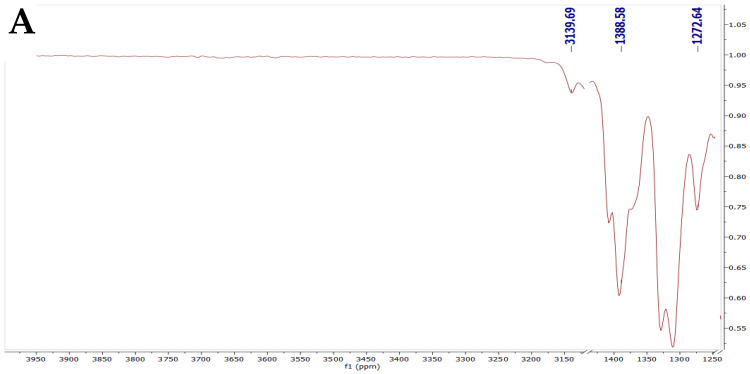

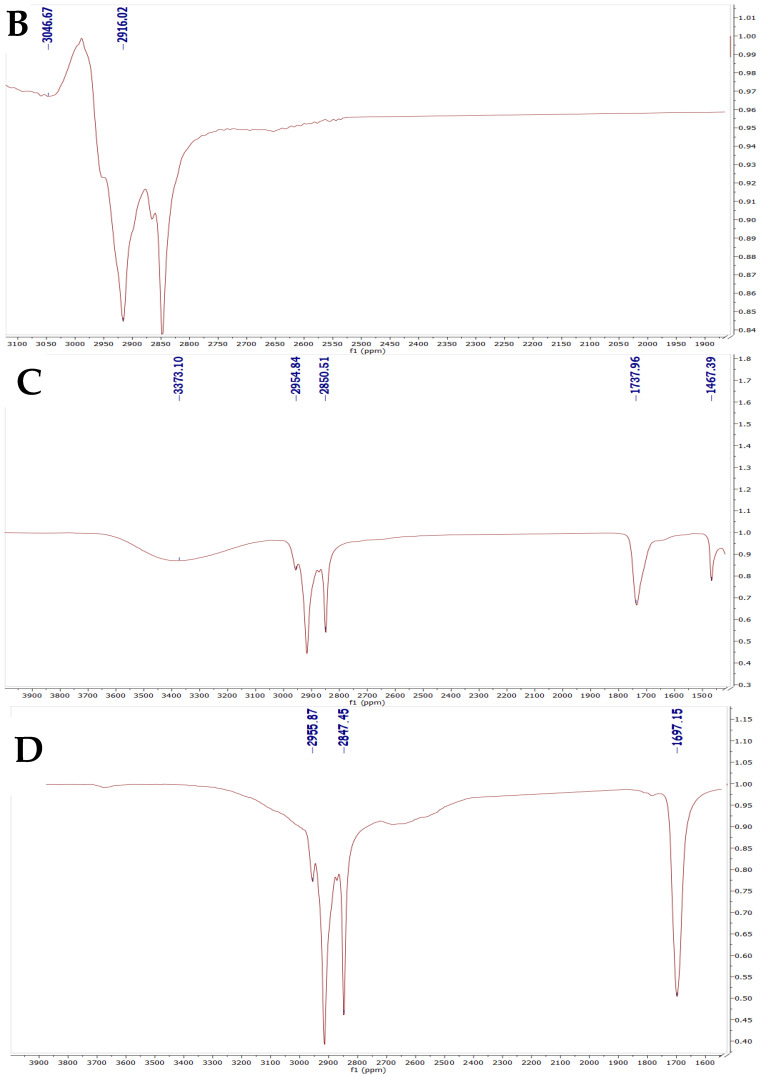

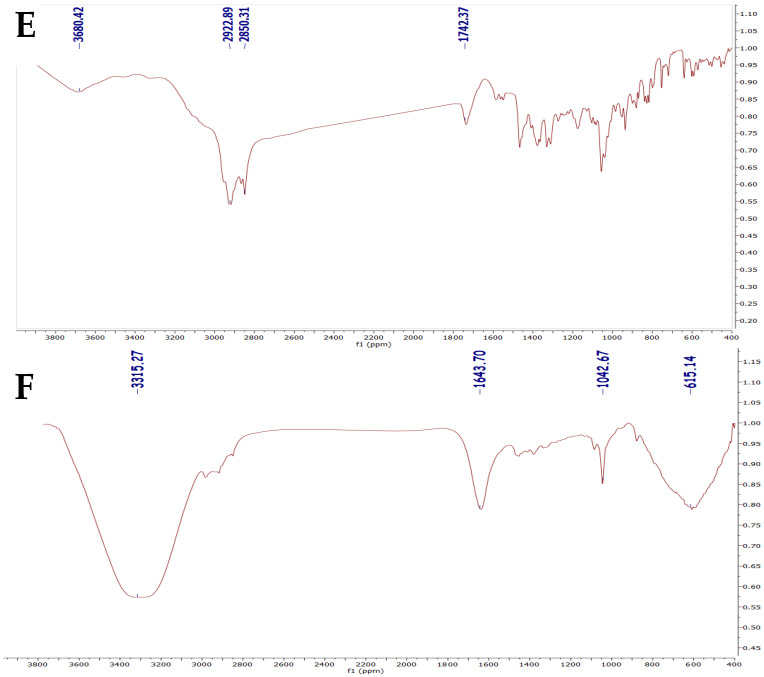
FTIR of (**A**) pure oxiconazole, (**B**) cholesterol, (**C**) span 60, (**D**) stearic acid, (**E**) physical mixture, (**F**) optimal oxiconazole-loaded novasomes.

The FTIR spectra, presented in Figure 5, revealed characteristic peaks for all individual components of the novasomal formulation, as well as for the physical mixture and the drug-loaded novasomes.

Oxiconazole spectra (Figure 5A) revealed an O-H stretching vibration at 3139 cm^−1^, a triazole ring peak at 1616 cm^−1^, CH_3_ extending at 1389 cm^−1^, and a CH_2_ group bending vibration at 1273 cm^−1^. Cholesterol spectra (Figure 5B) showed an O-H extending vibration at 3047 cm^−1^ and asymmetrical C-H bond extending at 2916 cm^−1^. Span 60 spectra (Figure 5C) displayed O-H extension at 3373 cm^−1^, aliphatic C-H extending at 2955 cm^−1^ and 2850 cm^−1^, CH_3_ group stretching at 1467 cm^−1^, and a characteristic peak for its cyclic five-membered ring at 1738 cm^−1^ [16]. Stearic acid spectra (Figure 5D) revealed characteristic peaks at 2955 cm^−1^ and 2847 cm^−1^ for CH_2_ stretching, with the C=O extension of the COOH group appearing at 1697 cm^−1^ [20]. The physical mixture spectra (Figure 5E) primarily exhibited the characteristic peaks of the excipients, with no notable shifts or new peaks predicting interaction. In contrast, the spectrum of the optimized oxiconazole-loaded novasomal formulation (Figure 5F) showed that certain characteristic peaks of oxiconazole either disappeared or were masked by the lipid peaks, suggesting successful entrapment of the drug within the lipid matrix. This observation confirms the successful oxiconazole loading into the novasomes and indicates compatibility between the drug and the polymeric components, as no significant chemical interactions were observed that would alter the distinct characteristic peaks of the drug or excipients [21,22].

### 2.6. Morphological Characterization of the Oxiconazole-Loaded Novasomes

**Figure 6:**
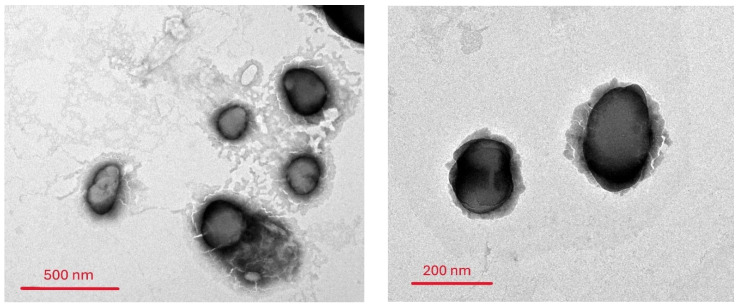
Morphology of vesicles with the optimal formula (R9).

Transmission electron microscopy (TEM) is employed to characterize the nanoparticle morphology, lamellarity, and size distribution [19]. Figure 6 displays representative TEM micrographs of the optimal novasomal formula vesicles (R9).

The images clearly show that the vesicles are nano-structured, spherically shaped, and unilamellar. Furthermore, they exhibit a uniform size distribution and a notable non-aggregating nature. This lack of aggregation can be directly correlated with the formulation’s high zeta potential (ZP) value, which promotes electrostatic repulsion between individual vesicles, thereby enhancing their colloidal stability [23].

### 2.7. Stability Evaluation for the Optimal Preparation

The physical stability of the optimized preparation was rigorously investigated by monitoring changes in its encapsulation efficiency (EE%), vesicle size (VS), zeta potential (ZP), and polydispersity index (PDI) during 3 and 6 months of storage at 4 °C (Table 3).

During the entire storage period, no aggregation or other abnormalities were observed. The analytical data indicated that the duration of storage revealed a statistically insignificant effect (*p* > 0.05) on the EE%, VS, ZP, PDI, and 8 h drug release (DR%) of the preparation.

### 2.8. Evaluation of Oxiconazole Novasomal Gel

The optimized gel formulation demonstrated excellent physical characteristics, appearing smooth and homogeneous. Its spreadability was measured at 7.3 ± 0.3 cm, indicating that the gel can be easily applied to the skin surface with minimal effort. The optimized formula has a pH value of 6.2 ± 0.2, falling within the acceptable physiological range for topical preparations, which is crucial for minimizing skin irritation. Finally, the optimal gel had a viscosity of 1890 ± 15 cp, ensuring adequate structural integrity and ease of application [22,24].

### 2.9. In Vitro Permeation of Oxiconazole from Prepared Novasomes

**Figure 7:**
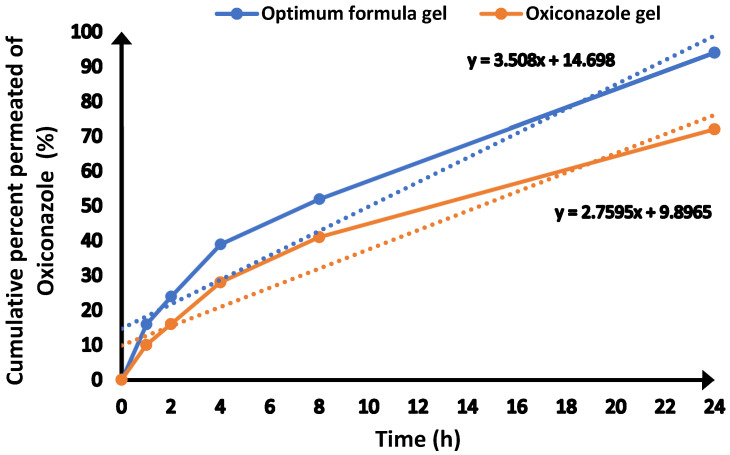
Permeation analysis of oxiconazole from optimized gel preparation and oxiconazole gel.

The permeation profiles of the optimized oxiconazole formulation gel and a plain oxiconazole gel were comparatively evaluated, as presented in Figure 7. For the optimized formulation, a significantly enhanced quantity of oxiconazole permeated within 24 h (1820 ± 35 µg/cm^2^) when compared to the oxiconazole gel (1440 ± 22 µg/cm^2^) (Table 4) (Figure 7).

Furthermore, the optimized formulation exhibited a Steady-State flux (J_ss_) of 3.508 g/cm^2^/h, whereas the oxiconazole gel yielded a Steady-State flux (J_ss_) of 2.759 g/cm^2^/h. These findings unequivocally highlight the superior permeation of oxiconazole when delivered via the optimized formulation.

### 2.10. In Vivo Assessment

**Figure 8:**
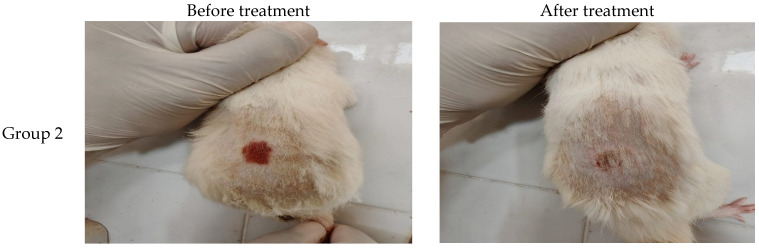

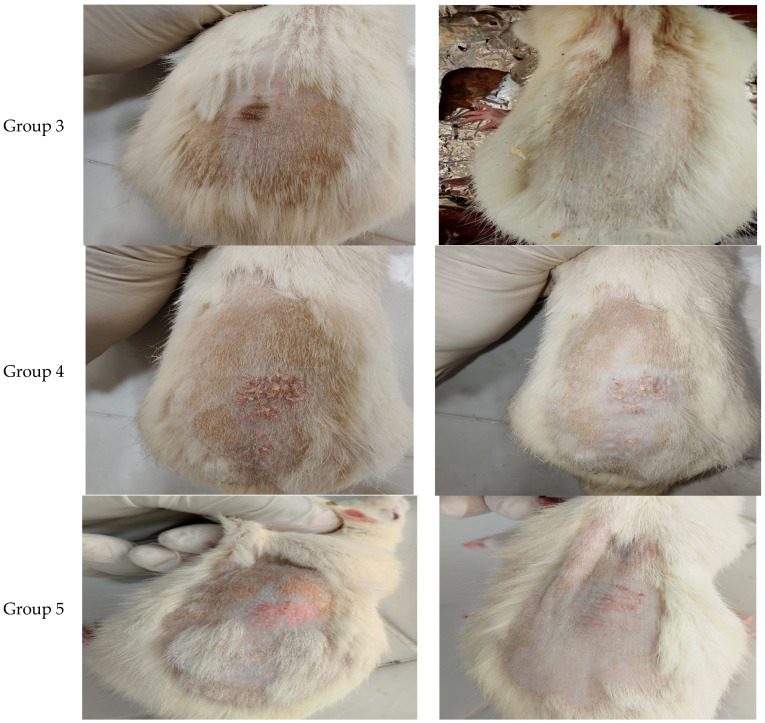
Skin specimens from various rats after induction Candida albicans and after therapy.

**Figure 9:**
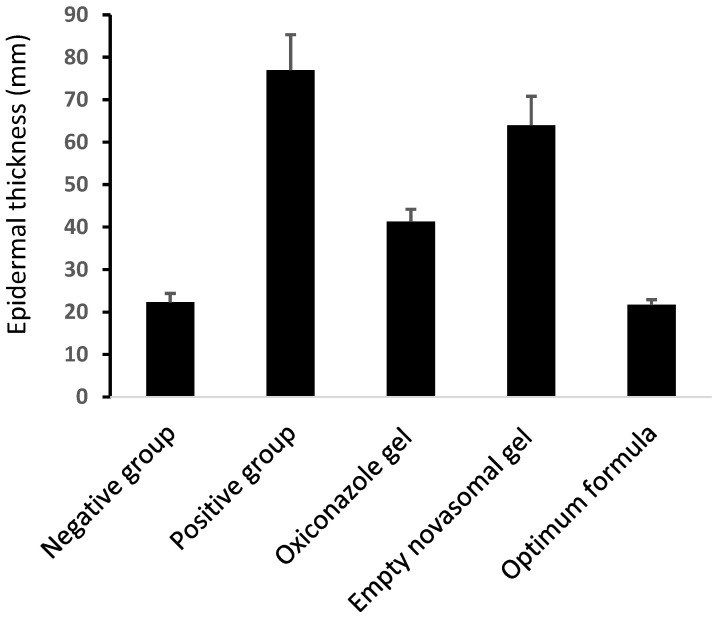
Epidermal thickness of skin sections in all groups.

Epidermal thickness was quantified by measuring six regularly spaced skin sections via ImageJ software (Version 1.53k NIH, Bethesda, MD, USA). For each tissue specimen, the average of these measurements was determined, and subsequently, the mean thickness for each experimental group was calculated (Figure 8 and Figure 9, and Table 5).

#### Histopathology Investigations

**Figure 10:**
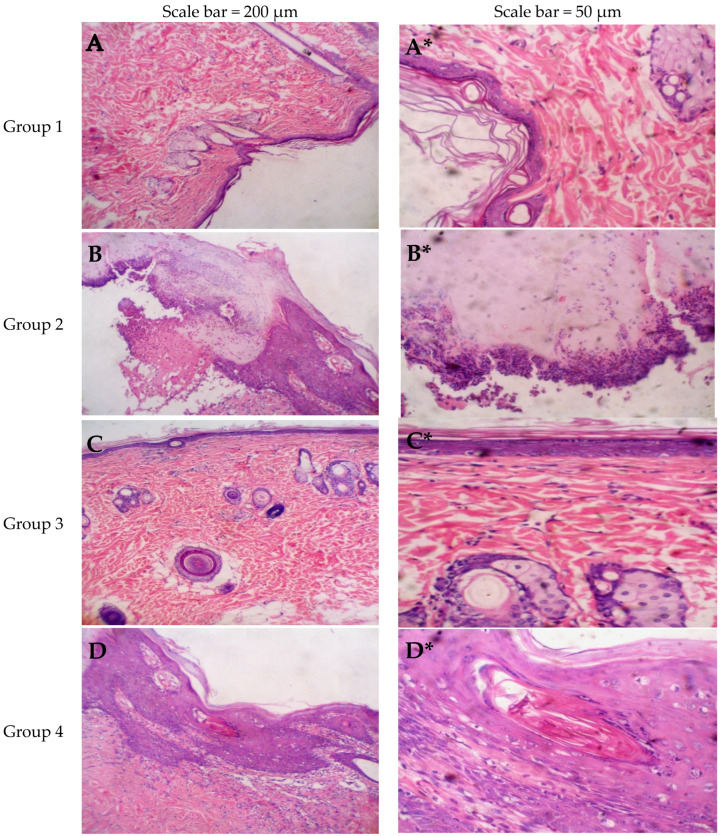

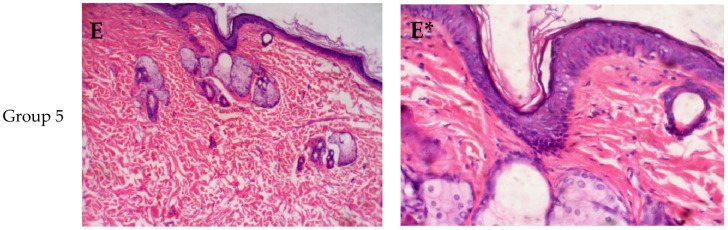
Images from histopathology investigations of skin tissues.

Figure 10A,A* images for negative control (Group 1) reveal uniform epidermal covering with no ulceration. The underlying dermis showed uniform dermal appendages with no inflammatory infiltrate and uniform collagen arrangement.

Figure 10B,B* images for positive control (Group 2) reveal acanthosis and compact hyperkeratosis with keratotic plug and focal ulceration with neutrophilic infiltrate. The underlying dermis showed diffuse chronic inflammatory cell infiltrate with foci of dermal edema. There is dermal collagenization and fibrosis.

Figure 10C,C* images for Oxiconazole gel without novasomal system (Group 3) show focal acanthosis and compact hyperkeratosis with keratotic plug, but with no ulceration. The underlying dermis shows uniform dermal appendages, no inflammatory infiltrate and uniform collagen arrangement.

Figure 10D,D* images for empty novasomal gel without oxiconazole (Group 4) exhibit thickening (acanthosis) and dense hyperkeratosis, characterized by keratotic plugs and localized ulceration associated with neutrophilic infiltration. The dermis beneath shows widespread infiltration of chronic inflammatory cells along with focal areas of interstitial edema. Furthermore, there is notable collagen deposition and fibrotic changes, reflecting ongoing chronic inflammatory processes and tissue remodeling.

Figure 10E,E* images for Oxiconazole novasomal gel (optimal formula) (Group 5) show continuous and intact surface without ulceration. The underlying dermis contains uniformly distributed dermal appendages, lacks inflammatory cell infiltration, and exhibits a consistent and orderly arrangement of collagen fibers.

Most importantly, the optimal formula demonstrated superior clinical efficacy against *Candida albicans* compared to both oxiconazole gel and other groups. This verifies that oxiconazole-loaded novasomes are promising vesicles for significantly enhancing oxiconazole’s antifungal potential.

## 3. Discussion

Stearic acid is extensively utilized in pharmaceutical formulations owing to its well-documented safety profile [25]. It has the ability to enhance the lipophilicity of the formulation’s matrix, which is advantageous for the encapsulation of the lipophilic oxiconazole [26]. The inherent lipophilicity of stearic acid stems from its prolonged (C_18_) and saturated alkyl chain. This structural feature contributes to an elevated phase transition temperature (Tc = 69 °C), promoting more rigid preparations and low-permeability vesicles, thereby leading to an increased EE% [16].

An elevated ratio or concentration of Span 60, characterized by its low HLB (4.7) because of the extended alkyl chain of its oleate moiety (C_18_), contributes to a reduction in the hydrophilicity of membrane pores and reduces membrane fluidity, leading to an increased EE% [27]. This can be related to the enhanced emulsification and stabilization provided by the lipid substance when a high surfactant concentration is present [28]. 

An elevation in the concentration of cholesterol results in a reduction in bilayer membrane permeability. This effect is attributed to the cholesterol-induced enhancement of membrane rigidity, which consequently improves the retention and encapsulation of the hydrophobic drug within the bilayer structure [29].

Increasing Span 60 concentration may be related to the increased drug encapsulation within the vesicles, potentially leading to the formation of gaps that widen the bilayers and consequently increase VS. Furthermore, the 18-carbon alkyl chain length of Span 60 might be involved in the formation of vesicles with a larger core space and a greater overall diameter [15,30].

Stearic acid, characterized by its elevated melting point (69 °C), contributes to increased melting viscosity, which in turn reduces the effectiveness of sonication in achieving smaller vesicle sizes [16]. 

Cholesterol was hypothesized to reduce vesicle size. This effect is attributed to cholesterol’s integration into the lipid bilayer, which enhances membrane rigidity and packing density. Consequently, this lowers the surface-free energy of the particles, leading to a reduction in vesicle size, particularly in formulations based on Span 60 [31].

Increasing both Span 60 and stearic acid simultaneously enhanced the hydrophobic domain of the bilayer, promoting greater entrapment of the lipophilic oxiconazole. However, the resulting thicker bilayer structure also led to larger vesicle size due to reduced membrane flexibility [12,17].

The elevation in negative charge can be attributed to the ionization of specific functional groups within these components. Stearic acid possesses a free carboxylic acid group (C_17_H_35_COOH), while both Span 60 (C_24_H_46_O_6_) and cholesterol (C_27_H_46_O) contain free hydroxyl groups in their molecular structures. During ionization, these functional groups contribute to the net negative charge on the vesicle surface, thereby influencing the electrostatic stability of the formulation. Consequently, the three parameters (stearic acid, Span 60, and cholesterol) revealed a positive and significant influence on ZP. The rigid, crystalline structure imparted by stearic acid further enhances membrane order and resistance to vesicle deformation, thereby improving the long-term stability by resisting the aggregation and fusion of nanovesicles [29,32].

The PDI assesses the breadth of the vesicle size (VS) distribution and, consequently, the overall homogeneity of vesicle sizes across the nanodispersion. A high PDI value signifies a heterogeneous distribution of vesicles, whereas a low PDI value reveals homogeneous, monodispersed vesicles [33].

Span 60, characterized by a low Hydrophilic–Lipophilic Balance (HLB ≈ 4.7), is inherently lipophilic. This property enhances its interaction with hydrophobic drug molecules. The increased rigidity imparted by Span 60 leads to reduced membrane permeability, thereby restricting the diffusion of encapsulated hydrophobic drugs and consequently slowing their release [17,34].

Stearic acid plays a critical function in modulating the drug release of hydrophobic compounds from novasomes due to its unique physicochemical characteristics. As a long-chain saturated fatty acid, stearic acid contributes to creating a more rigid as well as ordered bilayer structure within the vesicle membrane. This increased membrane rigidity, in turn, reduces the permeability of the bilayer, thereby slowing the diffusion of the encapsulated hydrophobic drug [35,36].

Cholesterol negatively impacted drug release from the novasomal vesicles. This effect is attributed to cholesterol’s ability to impart enhanced stability to the vesicles, leading to the formation of a more rigid and less permeable membrane. Such a robust membrane consequently reduces the outward diffusion of the entrapped drug from the novasomes, thereby slowing its release [37,38].

Increasing stearic acid in combination with cholesterol increased bilayer rigidity, reducing vesicular permeability and thereby enhancing drug entrapment while slowing drug release. This stabilizing effect of cholesterol–fatty acid interactions has been extensively documented for lipid vesicles, where cholesterol reduces bilayer fluidity and leakage [17].

The ZP of the optimal preparation was specifically evaluated as it is a critical parameter for evaluating the durability of colloidal nanoparticles. ZP quantifies the electrical charge present above the particles; higher absolute ZP findings signify a greater electrostatic repulsion between nanovesicles, thus contributing to a more stable nanovesicle system [39]. The optimized formulation exhibited a highly negative ZP, indicative of excellent dispersion quality. This negative surface charge on the novasomes is likely attributable to the incorporation of stearic acid, which enhances the stability of the novasomes in the aqueous phase [40].

The enhanced permeation observed with the formulated novasomes can likely be attributed to their small vesicle size (VS) [10,41].

The improved permeation observed can also be related to the inclusion of stearic acid. Saturated fatty acids, particularly those with a high melting point, have been shown to enhance permeation rates across biological membranes. Free fatty acids (FFAs) like stearic acid can rapidly incorporate into the lipid bilayers, increasing curvature stress that destabilizes the membrane structure, thereby increasing its permeability [25]. Furthermore, cholesterol functions as a penetration improver by facilitating the diffusion of the encapsulated drug through biological barriers [29,41].

Finally, the inclusion of ethanol further enhanced the drug’s permeation rate. Ethanol interacts with the polar head groups of stratum corneum (SC) lipids, lowering their melting point, and thus increasing lipid bilayer fluidity and cell-membrane permeability [42,43]. The observed maximum drug permeability from the vesicles is therefore attributed to a positive effect of ethanol, the particles themselves, and the SC lipid substances [44,45]. Additionally, Carbopol possesses excellent buffering capacity, which helps adjust the required pH and prevents skin irritation. When combined with nanocarriers, it contributes to achieving the desired viscosity and optimal bio-adhesion properties [46]. The observed electrostatic repulsion had TEER readings of over 30 ± 1.5k Ω, demonstrating acceptable skin integrity [47].

## 4. Materials and Methods

### 4.1. Materials

Oxiconazole nitrate was provided by Eva Pharma (Cairo, Egypt). Ethanol (99.9% *v*/*v*) and Carbopol 974P were obtained from Future Pharmaceutical Industries (Cairo, Egypt). Dialysis membranes (MWCO 14,000 Da) were purchased from SERVA Electrophoresis GmbH (Heidelberg, Germany). Phosphate-buffered saline (PBS, 10×) was acquired from Lonza (Verviers, Belgium). Stearic acid was supplied by ISOCHEM (Kochi, Kerala, India), Span 60 by Advent ChemBio (Navi Mumbai, Maharashtra, India), and cholesterol by Acros Organics (Geel, Belgium). All chemicals were analytical grade.

### 4.2. Experimental Design and Optimization:

A 3^3^ Box–Behnken design was applied to optimize oxiconazole-loaded novasomes, using Design-Expert^®^ software (Version 11; Stat-Ease Inc., Minneapolis, MN, USA) [45]. Three independent variables were selected: Span 60 (X_1_), stearic acid (X_2_), and cholesterol (X_3_). Their effects were evaluated on five dependent responses: encapsulation efficiency (Y_1_), vesicle size (Y_2_), zeta potential (Y_3_), polydispersity index (Y_4_), and percentage drug release after 8 h (Y_5_).

The design generated 17 runs, including five center points. Table 6 summarizes the variables and response constraints.

### 4.3. Preparation of Oxiconazole-Loaded Novasomes

Novasomes were prepared using the ethanol injection technique [8,10]. Oxiconazole (20 mg) and the specified quantities of Span 60, stearic acid, and cholesterol were dissolved in ethanol and maintained at 60 °C for complete solubilization.

Simultaneously, four times the ethanolic phase volume of PBS (pH 7.4) was preheated to 60 °C and stirred at 900 rpm. The ethanolic drug–lipid solution was injected slowly into the aqueous phase with continuous stirring. The appearance of turbidity indicated vesicle formation. Stirring continued for 2 h to ensure vesicle stabilization and size reduction. Formulations were stored at 4 °C until use.

### 4.4. Characterization of Novasomes

#### 4.4.1. Encapsulation Efficiency (EE%)

EE% was determined indirectly by measuring free drug in the supernatant after centrifugation at 20,000 rpm for 60 min at 4 °C (Sigma Laborzentrifugen GmbH, Osterode am Harz, Germany). The supernatant was diluted and analyzed at 229 nm using a UV–Vis spectrophotometer (Jasco, Hachioji, Tokyo, Japan) [8,10].

(6) $$EE\;\%=\frac{Total\;amount\;of\;Oxiconazole-Un\;entrapped\;Oxiconazole}{Total\;amount\;of\;Oxiconazole}\times 100$$

#### 4.4.2. Vesicle Size, Zeta Potential, and Polydispersity Index

Vesicle size (VS), zeta potential (ZP), and polydispersity index (PDI) were measured using dynamic light scattering (DLS) (Malvern Zetasizer Nano ZS, Malvern, UK) [13]. Prior to analysis, formulations were diluted with distilled water to reduce multiple scattering. Each sample was analyzed in triplicate.

#### 4.4.3. In Vitro Drug Release Study

Drug release was assessed using the dialysis bag diffusion method [48]. A volume equivalent to 2 mg oxiconazole was placed in pre-soaked dialysis bags (MWCO 14,000 Da). Bags were immersed in 40 mL PBS (pH 7.4) at 32 ± 0.5 °C and stirred at 100 rpm. Samples (1 mL) were withdrawn at 1, 2, 4, 8, and 24 h, replacing each with fresh medium. Oxiconazole content was measured spectrophotometrically at 229 nm.

(7) $$DR\%=\frac{The\;amount\;of\;Oxiconazole\;released\;at\;time\;t}{The\;preliminary\;amount\;of\;encapsulated\;oxiconazole}\times 100$$

### 4.5. Optimization of the Novasomal Formulation

Polynomial regression models were generated using Design-Expert^®^ to evaluate the influence of variables on responses and to construct 3D surface plots. Numerical optimization criteria aimed to maximize EE%, ZP, and DR% while minimizing VS and PDI. The formulation with the highest desirability factor was selected and prepared experimentally for validation [10,49].

### 4.6. FTIR Spectroscopy

FTIR spectra of oxiconazole, individual excipients, the physical mixture, and the optimized novasome formulation were recorded using ATR-FTIR spectroscopy (Shimadzu, Kyoto, Japan). Spectra were collected from 4000 to 400 cm^−1^ at 4 cm^−1^ resolution to assess possible interactions between drug and excipients [50].

### 4.7. Differential Scanning Calorimetry (DSC)

Thermal behavior of the optimized formulation and individual components was examined using DSC (Shimadzu DSC-60, Kyoto, Japan). Samples (5 mg) were sealed in aluminum pans and heated from room temperature to 250 °C at 10 °C/min under nitrogen (20 mL/min) [8,45].

### 4.8. Transmission Electron Microscopy (TEM)

Vesicle morphology was evaluated by TEM (TEM-1010, Tokyo, Japan). A small amount of gel containing the optimized formula was placed on a carbon-coated copper grid, stained with 1% phosphotungstic acid for contrast, blotted, air-dried, and examined [45].

### 4.9. In Vitro Skin Permeation Study

Rat abdominal skin was excised and mounted on vertical Franz diffusion cells (diffusion area 5 cm^2^). The receptor chamber contained PBS (pH 7.4) maintained at 37 ± 0.5 °C and stirred at 600 rpm [45,51]. Oxiconazole gel and optimized novasomal gel (equivalent to 2 mg drug) were applied to the donor side. Samples (1 mL) were collected at predetermined intervals and analyzed at 229 nm.

Steady-state flux (J_ss_) was obtained from the slope of the linear permeation phase [48].

K_p_ = j_ss_/C_o_ (8)

#### Skin Integrity (TEER)

To confirm membrane integrity, TEER was measured using an LCR bridge (Thurlby Thandar Instruments, Huntingdon, UK) at 1 kHz. Membranes showing resistance ≥1 kΩ were considered intact [47].

### 4.10. Preparation of Novasomal Gel

Carbopol 934 (1% *w*/*w*) was dispersed in distilled water containing benzalkonium chloride (0.01% *w*/*v*), glycerol (3% *w*/*v*), and propylene glycol (1% *w*/*v*). The optimized novasomal dispersion was incorporated with continuous stirring to achieve 1% *w*/*w* oxiconazole. Neutralization with 5% triethanolamine induced gel formation, which was allowed to equilibrate for 24 h at room temperature [3,45].

### 4.11. Evaluation of Gel Properties

#### 4.11.1. Homogeneity

A small quantity of gel was rubbed between the fingers to assess smoothness and uniformity [52].

#### 4.11.2. Spreadability

Approximately 0.5 g gel was placed between two glass plates, left for 5 min, and the diameter of spread measured [22,24].

#### 4.11.3. pH Measurement

One gram of gel was dispersed in 20 mL distilled water and measured using a calibrated pH meter (Jenway, Stone, UK), in triplicate [53].

#### 4.11.4. Viscosity

Viscosity was determined using a Brookfield R/S+ rheometer with CC14 spindle at 37 °C and 5 rpm for 10 s [22].

### 4.12. Stability Study

The optimized formulation was stored at 4 °C for 3 and 6 months. Vesicle size, EE%, ZP, PDI, and 8 h drug release were reassessed and compared to freshly prepared samples [17].

### 4.13. In Vivo Antifungal Study

#### 4.13.1. Animals and Ethics

Thirty albino rats were housed under controlled conditions (23 ± 5 °C). The protocol was approved by the Research Ethics Committee, Faculty of Pharmacy, Port Said University (PHARM.PSU.33).

#### 4.13.2. Immunosuppression

Cyclophosphamide was administered intraperitoneally: a loading dose of 75 mg/kg (3 days before infection), followed by two maintenance doses of 60 mg/kg every 4 days [21].

#### 4.13.3. Fungal Inoculum Preparation

*Candida albicans* ATCC 10231 was cultured on Sabouraud Dextrose Agar for 48 h at 30 °C, harvested, washed, and adjusted to 10^7^ CFU/mL [21].

#### 4.13.4. Infection Procedure

A 3 cm^2^ dorsal area was shaved and abraded gently, then inoculated with 0.1 mL fungal suspension. Occlusive dressing was applied for 48 h to establish infection [54].

Animals were divided into five groups (n = 6):

1. Negative control
2. Positive control (empty gel)
3. Oxiconazole gel
4. Empty novasomal gel
5. Optimized oxiconazole-loaded novasomal gel

#### 4.13.5. Histopathological Examination

Skin samples were fixed in 10% formalin, embedded in paraffin, sectioned at 3 µm, and stained with H&E and PAS. An independent pathologist assessed epidermal thickness, ulceration, inflammatory infiltrate, and collagen arrangement. Thickness measurements were quantified using ImageJ software [45,55].

### 4.14. Statistical Analysis

Data were expressed as mean ± SD (n = 3). One-way ANOVA was performed with significance at *p* < 0.05. Box–Behnken model statistics were generated using Design-Expert^®^ software (Version 11) [56].

## 5. Conclusions

Utilizing the ethanol injection method, oxiconazole-loaded novasome formulations were successfully prepared, and the R9 formula with Span 60 (75 mg), stearic acid (200 mg), and cholesterol (100 mg) was selected as an optimal formulation via a Box–Behnken design.

This optimized formulation displayed highly desirable attributes, including excellent encapsulation efficiency of 94.63 ± 1.60%, ideal vesicle size of 174 ± 1.15 nm, appropriate zeta potential of −46.5 ± 1.61 mV, a narrow polydispersity index of 0.184 ± 0.01, and prolonged drug release of 51 ± 0.50% within 8 h.

The optimized formulation was thoroughly characterized using FTIR, DSC, and TEM analyses, which confirmed the successful incorporation of oxiconazole into the novasomes and demonstrated compatibility between the drug and the polymeric constituents. The results further indicated that oxiconazole was uniformly distributed within the novasomes in an amorphous state, exhibiting a consistent particle size distribution and a distinct non-aggregating behavior.

The optimized formulation was incorporated into Carbopol 974 to generate the gel base, which was subsequently assessed both in vitro and in vivo to determine its permeation characteristics and therapeutic efficacy. The optimized gel exhibited a viscosity of 1890 ± 15 cp and a pH of 6.2 ± 0.2, yielding a homogeneous preparation with favorable spreadability. Enhanced permeation was achieved, with 94 ± 1.75% and a steady-state flux of 3.508 g/cm^2^/h over 24 h. Histopathological evaluation confirmed superior clinical performance of the optimized gel against Candida albicans, as evidenced by the absence of ulceration, inflammatory infiltrates, edema, and dermal fibroblastic proliferation, along with a minimal epidermal thickness of 21.7 ± 1.2 µm compared to oxiconazole gel and other groups.

As a result, the incorporation of oxiconazole into novasomes offers a highly promising and efficacious approach, holding significant value for the sustained and effective management of superficial fungal infections.

## Figures and Tables

**Table 1:** Experimental Design of Oxiconazole-Loaded Novasomes: Box–Behnken Design Parameters and Observed Responses.

Run	Independent Parameters			Dependent Parameters				
	X_1_ (mg)	X_2_ (mg)	X_3_ (mg)	Y_1_ (%)	Y_2_ (nm)	Y_3_ (mV)	Y_4_	Y_5_ (%)
R1	50	50	60	94.24 ± 3.80	246.2 ± 12.33	−61.6 ± 2.39	0.532 ± 0.02	62 ± 1.90
R2	75	200	20	90.37 ± 4.80	337.8 ± 14.50	−72.5 ± 4.30	0.375 ± 0.02	49 ± 2.10
R3	75	50	100	88.47 ± 4.60	374.4 ± 45.20	−64.6 ± 1.65	0.47 ± 0.08	42 ± 2.80
R4	50	125	100	93.45 ± 1.30	258.6 ± 4.30	−69.5 ± 1.10	0.34 ± 0.03	53 ± 2.0
R5 *	75	125	60	94.51 ± 2.50	235.3 ± 4.60	−26 ± 2.15	0.32 ± 0.02	55 ± 1.10
R6	50	200	60	91.9 ± 6.10	327.8 ± 17.80	−21.7 ± 0.20	0.358 ± 0.13	44 ± 1.60
R7 *	75	125	60	94.51 ± 2.50	235.3 ± 4.60	−26 ± 2.15	0.32 ± 0.02	55 ± 1.10
R8 *	75	125	60	94.51 ± 2.50	235.3 ± 4.60	−26 ± 2.15	0.32 ± 0.02	55 ± 1.10
R9	75	200	100	94.63 ± 1.60	174 ± 1.15	−46.5 ± 1.61	0.184 ± 0.01	51 ± 0.50
R10	50	125	20	92.8 ± 2.30	280.2 ± 62.79	−44.9 ± 0.90	0.383 ± 0.04	50 ± 0.80
R11	100	125	20	86.4 ± 4.80	387.3 ± 49.02	−73.8 ± 0.87	0.49 ± 0.14	35 ± 3.20
R12	100	200	60	90.1 ± 5.10	354 ± 11.80	−25.8 ± 0.20	0.408 ± 0.13	46 ± 1.50
R13	100	125	100	94.78 ± 1.40	209.1 ± 1.33	−38.2 ± 1.15	0.212 ± 0.04	48 ± 1.8
R14	75	50	20	92.94 ± 3.40	286.5 ± 35.96	−36.4 ± 0.97	0.197 ± 0.02	60 ± 1.50
R15 *	75	125	60	94.51 ± 2.50	235.3 ± 4.60	−26 ± 2.15	0.32 ± 0.02	55 ± 1.10
R16 *	75	125	60	94.51 ± 2.50	235.3 ± 4.60	−26 ± 2.15	0.32 ± 0.02	55 ± 1.10
R17	100	50	60	93.52 ± 2.10	253.2 ± 22.10	−33.5 ± 2.10	0.324 ± 0.04	53 ± 1.40

X_1_: span 60 concentration (mg); X_2_: stearic acid concentration (mg); X_3_: cholesterol concentration (mg); Y_1_: encapsulation efficiency percent; Y_2_: vesicle size (nm); Y_3_: zeta potential (mV); Y_4_: poly dispersity index; Y_5_: percent drug released within 8 h. Data revealed as the mean ± SD. * Represents the center points.

**Table 2:** Model evaluations for measured responses.

Response	Order	*p*-Value	F-Value	R^2^	Adeq. Precision	Significant Independent Parameters
EE %	Quadratic	<0.0001	61.98	0.991	24.723	X_1_, X_2_, X_3_
VS	Quadratic	<0.0178	7.83	0.933	9.497	X_2_, X_3_
ZP	Quadratic	<0.0036	15.78	0.966	10.954	X_1_, X_2_, X_3_
PDI	Quadratic	<0.0016	22.42	0.976	19.944	X_1_
DR% 8 h	Quadratic	<0.0001	276.89	0.998	59.256	X_1_, X_2_, X_3_

**Table 3:** The values for encapsulation efficiency (EE%), vesicle size (VS), zeta potential (ZP), polydispersity index (PDI), and 8 h drug release (DR% 8 h) for the optimal oxiconazole novasome preparation, when freshly prepared and during storage for 3 and 6 months at 4 °C.

Optimized Formula	Fresh Prepared	After 3 Months	After 6 Months
EE %	94.63 ± 1.6	93.3 ± 1.3	92.1 ± 2.4
PS (nm)	174 ± 1.15	174 ± 2.1	176 ± 2.7
ZP (mv)	−46.5 ± 1.61	−46 ± 1.1	−43.5 ± 3.3
PDI	0.184 ± 0.014	0.2 ± 0.02	0.173 ± 0.01
DR % 8 h	51 ± 0.50	49 ± 1.5	53 ± 1.2

**Table 4:** Skin permeation profiles of oxiconazole formulation gel and a plain oxiconazole gel within 24 h.

	The Permeated Amount of Oxiconazole (µg/cm^2^)	The Steady-State Flux (g/cm^2^/h)	The Cumulative Permeation Percentage (%)	The Permeability Coefficient (cm/h)
Optimal Gel Formula	1820 ± 35	3.508	94 ± 1.75	1.75 × 10^−3^
Oxiconazole Gel	1440 ± 22	2.759	72 ± 1.1	1.38 × 10^−3^

**Table 5:** Histopathological assessment of skin tissue sections in the studied groups.

Group	Group 1	Group 2	Group 3	Group 4	Group 5
Epidermal thickness (µm) (mean ± SD)	22.3 ± 2.1	77 ± 8.3	41.3 ± 2.9	64 ± 6.8	21.7 ± 1.2
Ulceration	Not Detected	Detected	Not Detected	Detected	Not Detected
Skin inflammatory infiltrate and edema	Not Detected	Detected	Not Detected	Detected	Not Detected
Dermal fibroblastic proliferation and collagen arrangement	No fibroblastic proliferation and uniform dermal collagen	There is fibroblastic proliferation and fibrosis	No fibroblastic proliferation and uniform dermal collagen	There is fibroblastic proliferation and fibrosis	No fibroblastic proliferation and uniform dermal collagen

**Table 6:** Assessment of the independent parameters in the Box–Behnken design.

Factor	Level		
Independent Parameters	Minimum (−1)	Moderate (0)	Maximum (1)
X_1_: Span 60 amount (mg)	50	75	100
X_2_: Stearic (mg)	50	125	200
X_3_: Cholesterol amount (mg)	20	60	100
Responses (dependent parameters)	limitations		
Y_1_: Encapsulation Efficiency (%)	Maximize		
Y_2_: Vesicle Size (nm)	Minimize		
Y_3_: Zeta Potential (mv)	Maximize		
Y_4_: polydispersity index	Minimize		
Y_5_: Percentage of drug release within 8 h (%)	Maximize		

## Data Availability

The original contributions presented in this study are included in the article. Further inquiries can be directed to the corresponding authors.

## Abbreviations

The following abbreviations are used in this manuscript:

VS	Vesicle Size
PS	Particle size
PDI	Polydispersity index
TLA	Zeta potential
EE	Entrapment (Encapsulation) efficiency
TEM	Transmission electron microscope
FTIR	Fourier Transform Infra-Red spectroscopy
DSC	Differential scanning calorimetry
PBS	Phosphate-buffered saline
DR	Drug release
J_ss_	Permeation flux value
C_0_	The preliminary drug concentration
HLB	Hydrophilic-lipophilic balance
DLS	Dynamic light scattering
K_P_	Permeability coefficient
Span 60	Sorbitan monostearate
SC	Stratum corneum
